# Mitochondrial Genomes of Two Barklice, *Psococerastis albimaculata* and *Longivalvus hyalospilus* (Psocoptera: Psocomorpha): Contrasting Rates in Mitochondrial Gene Rearrangement between Major Lineages of Psocodea

**DOI:** 10.1371/journal.pone.0061685

**Published:** 2013-04-22

**Authors:** Hu Li, Renfu Shao, Fan Song, Xuguo Zhou, Qianqian Yang, Zhihong Li, Wanzhi Cai

**Affiliations:** 1 Department of Entomology, China Agricultural University, Beijing, China; 2 GeneCology Research Centre, Faculty of Science, Education and Engineering, University of the Sunshine Coast, Maroochydore, Queensland, Australia; 3 Department of Entomology, University of Kentucky, Lexington, Kentucky, United States of America; University of Strasbourg, France

## Abstract

The superorder Psocodea has ∼10,000 described species in two orders: Psocoptera (barklice and booklice) and Phthiraptera (parasitic lice). One booklouse, *Liposcelis bostrychophila* and six species of parasitic lice have been sequenced for complete mitochondrial (mt) genomes; these seven species have the most rearranged mt genomes seen in insects. The mt genome of a barklouse, lepidopsocid sp., has also been sequenced and is much less rearranged than those of the booklouse and the parasitic lice. To further understand mt gene rearrangements in the Psocodea, we sequenced the mt genomes of two barklice, *Psococerastis albimaculata* and *Longivalvus hyalospilus*, the first representatives from the suborder Psocomorpha, which is the most species-rich suborder of the Psocodea. We found that these two barklice have the least rearranged mt genomes seen in the Psocodea to date: a protein-coding gene (*nad3*) and five tRNAs (*trnN*, *trnS1*, *trnE*, *trnM* and *trnC*) have translocated. Rearrangements of mt genes in these two barklice can be accounted for by two events of tandem duplication followed by random deletions. Phylogenetic analyses of the mt genome sequences support the view that Psocoptera is paraphyletic whereas Phthiraptera is monophyletic. The booklouse, *L. bostrychophila* (suborder Troctomorpha) is most closely related to the parasitic lice. The barklice (suborders Trogiomorpha and Psocomorpha) are closely related and form a monophyletic group. We conclude that mt gene rearrangement has been substantially faster in the lineage leading to the booklice and the parasitic lice than in the lineage leading to the barklice. Lifestyle change appears to be associated with the contrasting rates in mt gene rearrangements between the two lineages of the Psocodea.

## Introduction

The organization of mitochondrial (mt) genome is highly conserved in insects, as in most other bilateral animals [1], [2]. With few exceptions, the mt genomes of insects consist of 13 protein-coding genes, two rRNA genes, 22 tRNA genes and a large non-coding region (also called the control region) on a single circular chromosome [1]–[3]. Arrangement of genes in mt genomes is usually stable; most insects known retained exactly the ancestral pattern of mt gene arrangement or have minor changes from the ancestral pattern of mt gene arrangement [1], [4]–[8].

One group of insects that stands out and has major changes in the organization of mt genome is the superorder Psocodea. Psocodea has ∼10,000 described species in two orders: Psocoptera (barklice and booklice) and Phthiraptera (parasitic lice) [9]–[12]. Complete mt genomes have been sequenced for two species of the Psocoptera and six species of the Phthiraptera [13]–[20]. Compared to other insects, species of the Psocodea have three unusual features in their mt genomes that have not been found in any other insects. First, all of the eight species that have been sequenced have rearranged mt genomes. The booklouse, *Liposcelis bostrychophila*, and the six species of parasitic lice have the most rearranged mt genomes seen in insects - they differ at nearly every gene boundary from the putative ancestor of insects. Second, the booklouse, *L. bostrychophila* (Psocoptera: suborder Troctomorpha) and the parasitic lice in the suborder Anoplura have multipartite mt genomes. The mt genome of the booklouse, *L. bostrychophila*, has two mt chromosomes; each chromosome is 7–9 kb and has 16 to 22 genes [20]. The mt genomes of the human body louse, *Pediculus humanus*, and the human head louse, *Pediculus capitis*, have 20 minichromosomes; each minichromosome is 3–4 kb in size and contains one to three genes [14], [16]. The mt genome of the human pubic louse, *Pthirus pubis*, has at least 14 minichromosomes; each minichromosome is 1.8–2.7 kb in size and contains one to five genes [16]. Third, in the screamer louse, *Bothriometopus macrocnemis* (suborder Ischnocera), all mt genes are encoded by the same strand [18]. In the pigeon louse, *Campanulotes bidentatus compar* (suborder Ischnocera), all mt genes except *trnQ* are on the same strand [17].

The order Psocoptera has three suborders: Troctomorpha, Trogiomorpha and Psocomorpha. In addition to the booklouse, *L. bostrychophila* (suborder Troctomorpha), the complete mt genome of a barklouse, lepidopscocid sp. (suborder Trogiomorpha), has also been sequenced. The mt genome of the lepidopsocid sp. is much less rearranged than those of the booklouse and the parasitic lice; nevertheless, eight genes including a protein-coding gene have rearranged [13]. Prior to the present study, nothing is known about the mt genomes for species in the suborder Psocomorpha, which is the largest suborder of the Psocoptera, containing 25 of the 39 extant families and ∼4,000 of the ∼5,000 described species of the Psocoptera [10], [21], [22]. To further understand mt gene rearrangements and changes in mt genome organization in the Psocodea, we sequenced the mt genomes of two barklice, *Psococerastis albimaculata* and *Longivalvus hyalospilus*, the first representatives from the suborder Psocomorpha. We found that these barklice have the least rearranged mt genomes seen in the Psocodea to date. We show that there are contrasting rates in mt gene rearrangement between the two major lineages of the Psocodea.

## Materials and Methods

### Ethics Statement

No specific permits were required for the insects collected for this study. The insect specimens were collected from roadside vegetation by sweeping. The field collections did not involve endangered or protected species. The species in the family of Psocidae are common insects and are not included in the “List of Protected Animals in China”.

### Samples and DNA Extraction

Specimens of *P. albimaculata* and *L. hyalospilus* were collected in Kuankuoshui, Suiyang, Guizhou, China, in June 2010. Specimens were initially preserved in 95% ethanol in the field, and transferred to −20°C for long-term storage at the China Agricultural University (CAU). For each species, the genomic DNA was extracted from one male adult’s muscle tissues of the thorax using the DNeasy DNA Extraction kit (Qiagen).

### PCR Amplification and Sequencing

The mt genome was amplified by PCR in overlapping fragments with universal insect mt primers [23], and species-specific primers designed from sequenced fragments (Table S1). Short PCRs (<1.5 kb) were with Taq DNA polymerase (Qiagen); the cycling conditions were: 5 min at 94°C, followed by 35 cycles of 50 s at 94°C, 50 s at 48–55°C, 1–2 min at 72°C depending on the size of amplicons, and a final elongation step at 72°C for 10 min. Long PCRs (>1.5 kb) were with Long Taq DNA polymerase (New England BioLabs); the cycling conditions were: 30 s at 95°C, followed by 40 cycles of 10 s at 95°C, 50 s at 48–55°C, 3–6 min at 68°C depending on the size of amplicons, and a final elongation step at 68°C for 10 min. The concentration and size of PCR products were measured by spectrophotometry and agarose gel electrophoresis. PCR fragments were ligated into the pGEM-T Easy Vector (Promega); the resulting plasmid DNAs were isolated using the TIANprp Midi Plasmid Kit (Qiagen). All fragments were sequenced in both directions with an ABI 3730XL Genetic Analyzer, using the BigDye Terminator Sequencing Kit (Applied Biosystems) with two vector-specific primers and internal primers for primer walking.

### Assembly, Annotation and Bioinformatics Analysis

Sequence reads from the mt genome of each barklouse species were assembled into contigs with Sequencher (Gene Codes). Protein-coding genes and rRNA genes were identified by BLAST searches in GenBank and then confirmed by alignment with homologous genes from other insects. tRNA genes were identified with tRNAscan-SE v.1.21 [24]. *trnR and trnS1,* which could not be identified by tRNAscan-SE, were determined by sequence similarity comparison with tRNA genes of other insects. The base composition, codon usage, and nucleotide substitution were analyzed with Mega 5.0 [25]. Secondary structures of stem-loop in control region were folded using Mfold [26].

### Sequence Alignment

Four species from the Psocoptera and six species from the Phthiraptera were included in our phylogenetic analyses (Table 1). These species are: 1) three barklice, *P. albimaculata*, *L. hyalospilus* and lepidopsocid sp. [13]; 2) a booklouse, *L. bostrychophila* [20]; 3) four chewing lice, *Bothriometopus macrocnemis* [18], *Campanulotes bidentatus compar* [17], *Ibidoecus bisignatus* [19], and *Heterodoxus macropus* [15]; 4) the human body louse, *Pediculus humanus* [14]; and 5) the human pubic louse, *Pthirus pubis* [16]. Two true bugs, *Alloeorhynchus bakeri* and *Halyomorpha halys* (Hemiptera) [27], [28], the lacewing, *Chrysoperla nipponensis* (Neuroptera) [29], and the ground beetle, *Calosoma* sp. (Coleoptera) [30], were used as outgroups.

**Table 1:** Species of insects used in the phylogenetic analyses in the present study.

Order/suborder	Family	Species	Accessionnumber	Reference
Psocoptera				
Trogiomorpha	Lepidopsocidae	lepidopsocid sp.	NC_004816	[13]
Psocomorpha	Psocidae	*Psococerastis albimaculata*	JQ910989	present study
		*Longivalvus hyalospilus*	JQ910986	present study
Troctomorpha	Liposcelidae	*Liposcelis bostrychophila*	JN645275JN645276	[20]
Phthiraptera				
Ischnocera	Philopteridae	*Bothriometopus macrocnemis*	NC_009983	[18]
		*Campanulotes bidentatus compar*	NC_007884	[17]
		*Ibidoecus bisignatus*	NC_015999	[19]
Amblycera	Boopidae	*Heterodoxus macropus*	NC_002651	[15]
Anoplura	Pediculidae	*Pediculus humanus*	FJ499473–90	[14]
		*Pthirus pubis*	EU219987–95,HM241895–8	[16]
Hemiptera				
	Nabidae	*Alloeorhynchus bakeri*	NC_016432	[28]
	Pentatomidae	*Halyomorpha halys*	NC_013272	[27]
Neuroptera				
	Chrysopidae	*Chrysoperla nipponensis*	NC_015093	[29]
Coleoptera				
	Carabidae	*Calosoma* sp.	NC_018339	[30]

Sequences of all mt protein-coding genes and rRNA genes except *nad4* were used in phylogenetic analyses; *nad4* was excluded because it was not identified in the human pubic louse, *P. pubis* [16]. Segments of identical sequences (26–127 bp long) shared between five pairs of mt genes in the human body louse, *P. humanus*, and the human pubic louse, *P. pubis* [14], [16], were also excluded to ensure only homologous regions of the mt genes were aligned and used in subsequent phylogenetic analyses. Alignment of the nucleotide sequences of each protein-coding gene and its putative amino acid sequence was with MUSCLE [31], adjusted to preserve the reading frame. Sequences of each rRNA gene were aligned with the GUIDANCE algorithm [32], 33, adjusted to its RNA secondary structure [34]. The alignments of individual genes were concatenated after removing poorly aligned sites using Gblocks 0.91 [35].

### Phylogenetic Analysis

Three alignments were used for phylogenetic analyses: 1) a concatenated nucleotide sequence alignment of protein-coding genes and two rRNA genes (PCG123R); 2) a concatenated nucleotide sequence alignment of the first and the second codon positions of protein-coding genes and two rRNA genes (PCG12R); and 3) a concatenated amino acid sequence alignment of protein-coding genes (AA). Partitioned ML and Bayesian analyses were run with PCG123R, PCG12R and AA matrix, using RAxML 7.0.3 [36] and MrBayes 3.2.1 [37]. The best-fit model for the amino acid sequence alignment was determined with ProtTest [38], and the jModelTest 0.1.1 [39] was used for the nucleotide sequence of each gene, according to the Akaike Information Criterion (AIC). For the ML analyses, GTRMIX option for nucleotide sequence and MtREV model for amino acid sequence were used to optimize the topology. For the combined dataset, 1,000 independent runs from random starting trees were performed to find the highest scoring replicate. Node support was calculated by acquiring bootstrap values from heuristic searches of 1000 resampled datasets, using the rapid bootstrap feature of RAxML [40].

For Bayesian analyses, the most appropriate substitution model was GTR+I+G model for each protein-coding gene, 1st and 2nd codon positions of protein-coding genes, and *rrnL* gene; GTR+G model for *rrnS* gene; and MtREV model for amino acid sequence of each protein-coding genes. Two simultaneous runs of 10 million generations were conducted for the matrix and trees were sampled every 1,000 generations, with the first 25% discarded as burn-in. Stationarity was considered to be reached when the average standard deviation of split frequencies was below 0.01 [41].

## Results and Discussion

### Mitochondrial Genomes of the Barklice, *Psococerastis Albimaculata* and *Longivalvus Hyalospilus*

**Figure 1:**
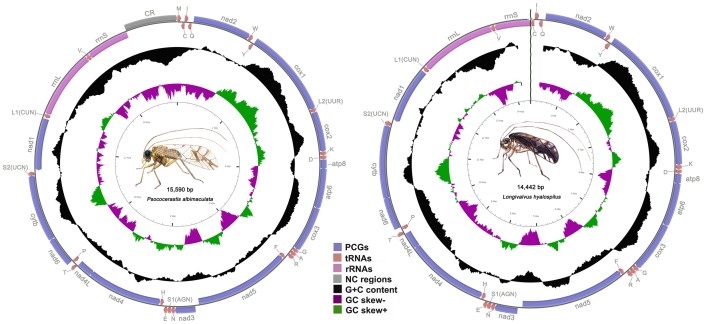
Mitochondrial genomes of the barklice, *Psococerastis albimaculata* and *Longivalvus hyalospilus*. Circular maps were drawn with CGView [42]. Arrows indicate the orientation of gene transcription. Protein-coding genes are shown as blue arrows, rRNA genes as purple arrows, tRNA genes as brown arrows and large non-coding regions (>100 bp) as grey rectangle. Abbreviations of gene names are: *atp6* and *atp8* for ATP synthase subunits 6 and 8, *cox1*–*3* for cytochrome oxidase subunits 1–3, *cytb* for cytochrome *b*, *nad1*–*6* and *nad4L* for NADH dehydrogenase subunits 1–6 and 4L, *rrnL* and *rrnS* for large and small rRNA subunits. tRNA genes are indicated with their one-letter corresponding amino acids; the two tRNA genes for leucine and serine have different anticodons: *L1* (anticodon TAG), *L2* (TAA), *S1* (TCT) and *S2* (TGA). The GC content is plotted using a black sliding window, as the deviation from the average GC content of the entire sequence. GC-skew is plotted as the deviation from the average GC-skew of the entire sequence. The inner cycle indicates the location of genes in the mt genome.

The mt genome of *P. albimaculata* contains the entire set of 37 genes (13 protein-coding genes, 22 tRNA genes, and two rRNA genes; Figure 1 and Table S2) and a putative control region that are usually present in animal mt genomes [1], [3]. We found the same set of mt genes in *L. hyalospilus*, except the control region and the adjacent *trnM* gene due to our unsuccess to amplify this region (Figure 1 and Table S3). These two barklice have the same arrangement of mt genes to each other but differs from the putative ancestor of insects: a protein-coding gene (*nad3*) and five tRNA genes (*trnN, trnS1, trnE, trnM and trnC*) have translocated. Genes are encoded by both strands in the mt genomes of these two barklice: 14 genes on one strand whereas the rest on the other strand (Table S2 and S3). Thirteen pairs of adjacent genes in the mt genomes of these two barklice overlap by 1 to 16 bp. All of the protein-coding genes of the two barklice start with ATN codons and stop with TAA/TAG codons or truncated codons TA or T.

The nucleotide compositions of the mt genomes of the two barklice are biased toward A and T. The nucleotide skew statistics for the entire majority strand indicate moderate A skew and obvious C skew, and the coding strand of protein-coding genes display a moderate T skew and slight C skew (Table 2). The A+T-richness of mt genomes of these two barklice is reflected further in the codon usage (Table S4). The overall ratio of G+C-rich codons to A+T-rich codons is 0.13 in the two barklice and there is a strong bias to A+T at the third codon positions of the protein-coding genes (Table 3).

**Table 2:** Codon usage of the protein-coding genes in the mitochondrial genomes of *Psococerastis albimaculata* and *Longivalvus hyalospilus*.

	*P. albimaculata*		*L. hyalospilus*	
Codon	count	%	count	%
A+T-rich codons^a^	1482	40.0	1509	41.0
G+C-rich codons^b^	473	12.8	480	13.0
Codon ratio^c^	0.13		0.13	

a A+T-rich codons are those encoding amino acids Asn, Ile, Lys, Met, Phe, and Tyr.

b G+C-rich codons are those encoding amino acids Ala, Arg, Gly, and Pro.

c Codon ratio is G+C-rich codons/A+T-rich codons.

**Table 3:** Nucleotide composition at each codon position of the protein-coding genes in the mitochondrial genomes of *Psococerastis albimaculata* and *Longivalvus hyalospilus.*

	AT %		GC %		AT-skew		GC-skew	
Codon	Pa	Lh	Pa	Lh	Pa	Lh	Pa	Lh
All codon	75.0	72.3	25.0	27.7	−0.14	−0.14	−0.03	−0.05
1st position	69.8	68.2	30.2	31.8	−0.009	0.005	0.20	0.16
2nd position	68.3	68.0	31.7	32.0	−0.39	−0.40	−0.11	−0.13
3rd position	87.0	80.6	13.0	19.4	−0.05	−0.03	−0.35	−0.25

Pa, *P. albimaculata*; Lh, *L. hyalospilus*; AT-skew = (A−T)/(A+T); GC-skew = (G−C)/(G+C).

**Figure 2:**
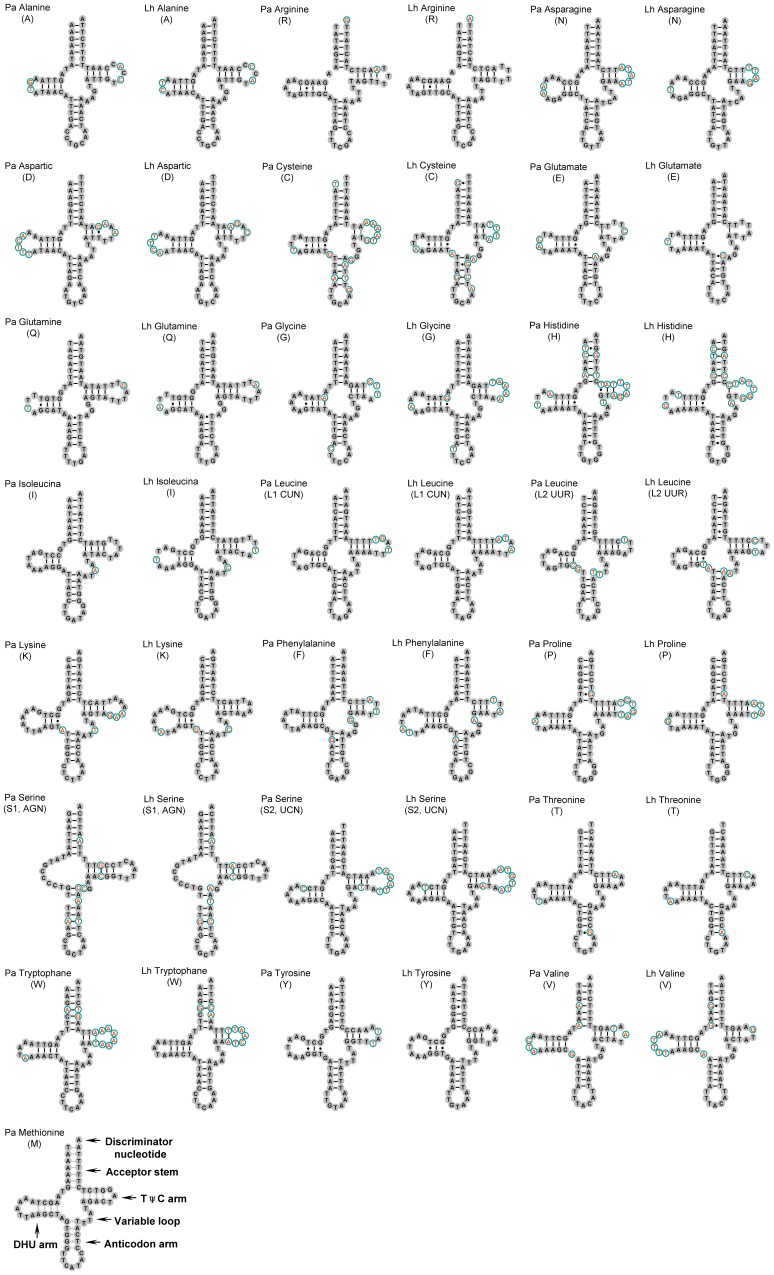
Inferred secondary structure of the mitochondrial tRNAs of the barklice, *Psococerastis albimaculata* and *Longivalvus hyalospilus*. tRNAs are labeled with the abbreviations of their corresponding amino acids. Lines indicate Watson-Crick bonds; dots indicate GU bonds. Green circle with red letter inside highlight variations of nucleotides in tRNA between two barklice.

The multiple alignment of 21 tRNA genes (excluded *trnM*) extends over 1,366 positions and contains 1,229 conserved nucleotides (90.0%) between the two barklice. Nucleotides at the stems and anticodon loops were conserved; variations were largely restricted to the loop of TψC and DHU arm (Figure 2). The nucleotide sequences of the mt genomes of two barklice, *P. albimaculata* and *L. hyalospilus*, have been deposited in GenBank under accession numbers JQ910989 and JQ910986.

### The Control Region in the Mitochondrial Genome of the Barklouse, *Psococerastis albimaculata*

**Figure 3:**
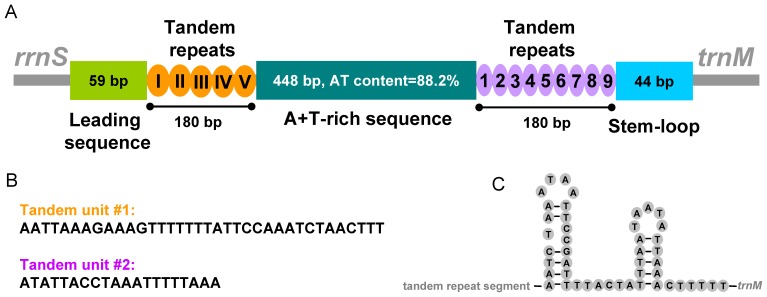
The control region in the mitochondrial genome of the barklouse, *Psococerastis albimaculata*. A) Structural elements in the control region; B) sequences of the tandem-repeat units; C) predicated stem-loop structures.

The putative control region (911 bp) was flanked by *rrnS* and *trnM* in the mt genome of *P. albimaculata*. The control region is highly AT-rich (86.9%; majority strand) and can be divided into five parts (Figure 3A): 1) 59 bp leading sequence; 2) 180 bp tandem repeat sequences consisting of five 36 bp repeat units (Figure 3B); 3) 448 bp A+T-rich sequences (A+T 88.2%); 4) 180 bp tandem repeated sequences consisting of nine 20 bp repeat units (Figure 3B); and 5) 44 bp stem-loop structure (Figure 3C). Stem-loops are common in the control regions of animal mt genomes and may have roles in the initiation of gene transcription and DNA replication [43]–[47]. The pattern of two tandem repeated sequences with an A+T-rich sequence in between is also present in the control region of the other barklouse, lepidopsocid-RS (Trogiomorpha), but not in the booklouse, *L. bostrychophila* (Troctomorpha), nor in any parasitic lice.

Outside the control region, there are 146 bp non-coding sequences in 13 intergenic regions of *P. albimaculata*, and 178 bp non-coding sequences in 12 intergenic regions in *L. hyalospilus*. Most non-coding sequences are scatted in short runs (1–16 bp in *P. albimaculata* and 1–26 bp in *L. hyalospilus*). However, two of these non-coding sequences are longer in length and locate in the same intergenic regions in the two barklice: 1) between *trnQ* and *nad2* (29 bp in *P. albimaculata* and 38 bp in *L. hyalospilus*); and 2) between *nad5* and *nad3* (71 bp in *P. albimaculata* and 73 bp in *L. hyalospilus*). These two non-coding sequences are in the regions where gene rearrangement occurred and are likely generated from events of tandem duplication followed by random deletions (See below).

### Phylogenetic Relationships among Major Lineages of the Psocodea Inferred from Mitochondrial Genome Sequences

The Psocoptera (booklice and barklice) and the Phthiraptera (parasitic lice) have traditionally been recognized as two separate orders [12], [48], [49]. The Psocoptera (booklice and barklice) are free-living insects and consist of over 5,000 species with a world-wide distribution, and are divided into three suborders: Trogiomorpha, Troctomorpha and Psocomorpha [10], [22]. Members of the order Phthiraptera (∼4,900 species) are wingless insects, parasitic on birds and mammals. There are four recognized suborders in the Phthiraptera: Amblycera, Ischnocera, Anoplura and Rhynchophthirina [9], [11], [50]. Both morphological and molecular analyses indicate a close relationship between parasitic lice (Phthiraptera) and booklice (family Liposcelididae); the order Psocoptera is therefore paraphyletic [9], [12], [20], [51]–[55]. The monophyly of the Phthiraptera, however, has been controversial. A number of shared morphological and physiological characters support the monophyly of the Phthiraptera [9], [48], [49]. Analyses of mt 12S and 16S rDNA and nuclear 18S rDNA sequences, however, indicate that the parasitic lice are paraphyletic: the suborder Amblycera is more closely related to the booklouse family Liposcelididae than to the other three suborders of the parasitic lice [12], [51], [54].

**Figure 4:**
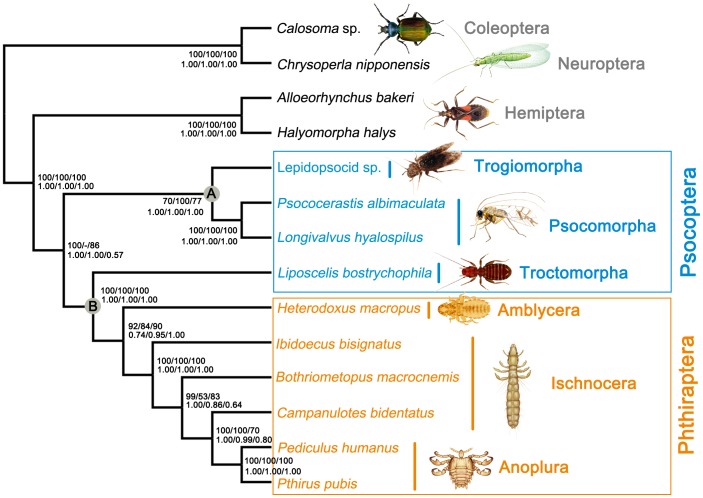
Phylogenetic relationships among major lineages of the Psocodea inferred from mitochondrial genome sequences. Numbers close to the branching points are ML bootstrap support values (above) and Bayesian posterior probabilities (below) in percentages; only support above 50% is shown. Numbers from left to right are from PCG123R, PCG12R and AA alignments respectively.

We tested the phylogenetic relationships among the major lineages of the Psocodea with the mt genome sequences of *P. albimaculata* and *L. hyalospilus*, and eight other species of the Psocodea. We used three different datasets: 1) a concatenated nucleotide sequence alignment of protein-coding genes and two rRNA genes (PCG123R); 2) a concatenated nucleotide sequence alignment of the first and the second codon positions of protein-coding genes and two rRNA genes (PCG12R); and 3) a concatenated amino acid sequence alignment of protein-coding genes (AA). All three datasets were run with partitioned ML and Bayesian analyses based on best-fit models. We recovered two major clades in the Psocodea with strong support regardless the dataset and the method we used: 1) species of barklice in the suborders Psocomorpha and Trogiomorpha were clustered together (Clade A, Figure 4); and 2) the booklouse, *L. bostrychophila* (suborder Troctomorpha), formed a clade with the parasitic lice (Clade B, Figure 4). The parasitic lice (Phthiraptera) are monophyletic with strong support; within the parasitic lice, the suborder Ischnocera, however, is paraphyletic.

### Contrasting Rates in Mitochondrial Gene Rearrangement between Two Major Lineages of the Psocodea

Seven species from the Clade B above have been sequenced for complete mt genomes previously, i.e., the booklouse, *L bostrychophila* (suborder Troctomorpha) [20] and six parasitic lice [14]–[19]. All of these seven species have extremely rearranged mt genomes, having little similarity in gene arrangement with each other, nor with any other insects. Both translocations and inversions occurred in these species relative to the ancestral gene arrangement of insects. Only four ancestral gene boundaries of insects (*trnG*-*nad3*, *trrnL1*-*rrnL*, *nad4*-*nad4L*, and *atp8*-*atp6*) were found in the Clade B species, of which only *atp8*-*atp6* was retained by all of the seven species in the Clade B. Apparently, mt gene rearrangement has been occurring much more often in the Clade B species than in other insects.

**Figure 5:**
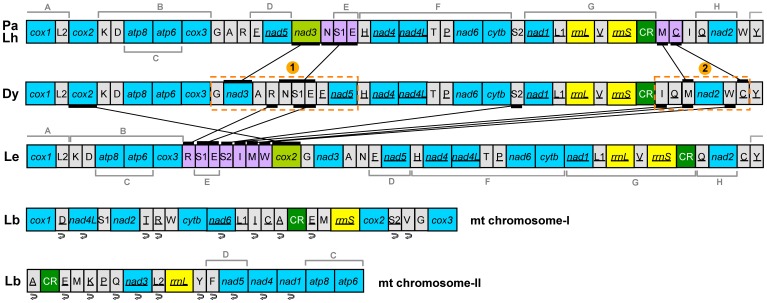
Comparison of mitochondrial gene arrangement between barklice, booklice and the hypothetical ancestor of insects. Abbreviations of species names are: Pa for *P. albimaculata*, Lh for *L. hyalospilus*, Dy for *Drosophila yakuba*, Le for lepidopsocid, and Lb for *L. bostrychophila*. Abbreviations of gene names follow Figure 1. Genes are transcribed from left to right except those underlined, which have the opposite transcriptional orientation. Pa and Lh have the same set of mt genes and gene arrangement, except the control region and the adjacent *trnM* gene due to our unsuccess to amplify this region in Lh. Shared gene boundaries are indicated by brackets and letter codes: A) *trnY*-*cox1*-*trnL2*; B) from *trnK* to *cox3*; C) *atp8*-*atp6*; D) *trnF*-*nad5*; E) *trnS1*- *trnE*; F) from *trnH* to *cytb*; G) from *nad1* to *CR*; and H) *trnQ*-*nad2*. Two ancestral gene blocks highlighted by the orange; broken lines indicate the inferred blocks that had duplicated in tandem. The circling arrows indicate inversions of mt genes relative to the hypothetical ancestor of insects.

**Figure 6:**
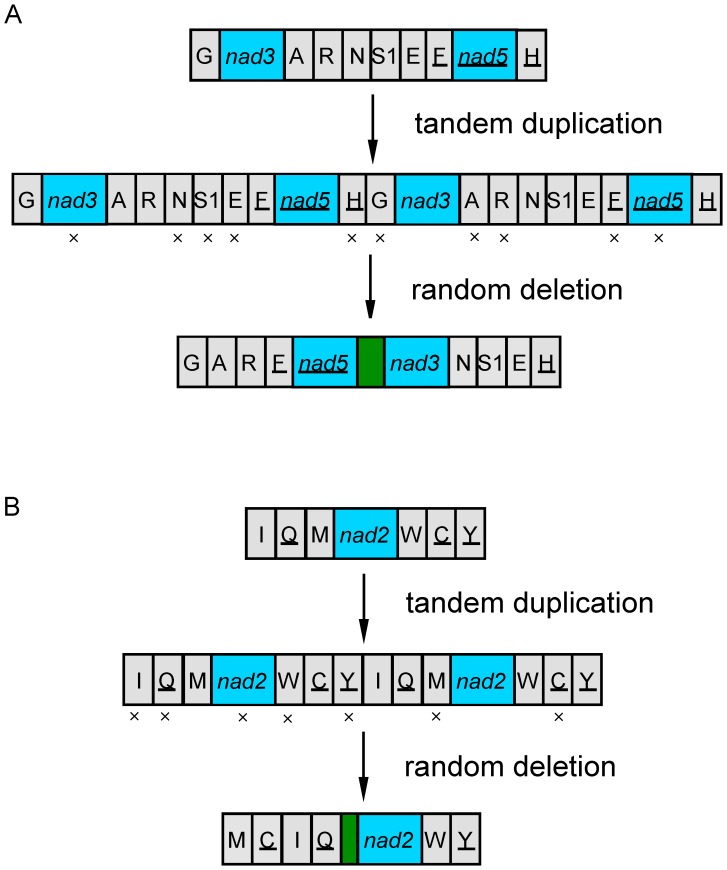
Inferred tandem-duplication-and-random-deletion events that account for the mitochondrial gene rearrangements in the barklice, *Psococerastis albimaculata* and *Longivalvus hyalospilus*. A) genes between *cox3* and *nad4*; B) genes between *CR* and *cox1*. Two longer non-coding sequences are highlighted by green color.

In contrast, species from the Clade A investigated to date have much less rearranged mt genomes and retained most of the ancestral gene arrangements of insects [13]. In particular, the two barklice we sequenced in the present study, *P. albimaculata* and *L. hyalospilus*, have the least rearranged mt genomes seen in the Psocodea: a protein-coding gene (*nad3*) and five tRNAs (*trnN*, *trnS1*, *trnE*, *trnM* and *trnC*) have translocated relative to that of the ancestor of insects (Figure 5). Gene rearrangement in these two barklice occurred in the two “hot spot” regions for gene rearrangement [56]: 1) between *cox3* and *trnH*, and 2) between *CR* and *trnY* (Figure 5). Two events of tandem duplication followed by random deletions could account for, straightforwardly, the rearrangement of mt genes observed in *P. albimaculata* and *L. hyalospilus* (Figure 6). The number of mt genes that have rearranged in *P. albimaculata* and *L. hyalospilus* is even less than in the other barklouse, lepidopsocid-RS (*cox2* and seven tRNAs rearranged) [13]. Together, 20 gene boundaries (Figure 5, seven gene blocks: A, B, D, E, F, G, and H) are shared and 32 ancestral gene boundaries are retained in these three barklice.

Tandem duplication followed by random deletion model has been proposed to account for mt gene rearrangement [8], [57]–[59]. This model can explain the mt gene rearrangements observed in the two barklice, *P. albimaculata* and *L. hyalospilus* (Figure 6). The rearranged mt gene blocks of these two barklice, one from *trnG* to *trnH* and another from *trnM* to *trnY* can be generated by a single event of tandem duplication of the ancestral gene block from *trnG* to *trnH* (Figure 6A) and from *trnI* to *trnY* (Figure 6B), respectively, and followed by random deletion of excess genes. The non-coding sequences present in the two rearranged gene boundaries (between *trnQ* and *nad2*, and between *nad5* and *nad3*) are likely traces of the random deletion. The mt gene rearrangement in the other barklouse lepidopsocid-RS (suborder Trogiomorpha) is more complicated than in *P. albimaculata* and *L. hyalospilus*; *cox2* gene and four tRNA genes have translocated from long distance and cannot be accounted for alone by tandem duplication followed by random deletion model. The mt gene rearrangements in the booklice and parasitic lice are much more complicated than in the barklice; multiple mechanisms and frequent rearrangement events are likely involved [16], [60], [61].

What caused the substantial difference between the two clades of the Psocodea in the rates of mt gene rearrangement? An obvious difference between the two clades is the lifestyle. The barklice in the Clade A are entirely free-living insects, which often feed on fungal spores; whereas Clade B (booklice and parasitic lice) is a mixture of short-term commensal and parasitic. The booklouse, *L. bostrychophila*, is mainly an inhabitant of households and a major pest to stored grains world-wide; moreover, there are many records of various species of booklice in the plumage of birds and the pelage of mammals, as well as in their nests [62], [63]. This association is believed to be a short-term commensal (non-parasitic) relationship, which may have given rise to a parasitic and permanent association [64]. All parasitic lice (Phthiraptera) feed on the skin, skin debris or blood of their vertebrate hosts and spend their entire life cycle on the body of the host [12]. The lifestyle change in the Clade B appears to be associated with an increased rate of mt gene rearrangement. However, why they are linked and exactly what biological and lifestyle factors contributed to the contrasting rates in mt gene rearrangement between the two major lineages of the Psocodea are not yet clear to us and remains to be determined.

## Supporting Information

Table S1 **Primers used in the present study.**
(DOC)

Table S2 **Genes in the mitochondrial genome of the barklouse,** ***Psococerastis albimaculata.***
(DOC)

Table S3 **Genes in the mitochondrial genome of the barklouse,** ***Longivalvus hyalospilus.***
(DOC)

Table S4 **Codon usage in the protein-coding genes of the mitochondrial genomes of the barklice,** ***Psococerastis albimaculata*** **and** ***Longivalvus hyalospilus.***
(DOC)
