# Supplementary material for: Mitochondrial Genomes of Two Barklice, Psococerastis albimaculata and Longivalvus hyalospilus (Psocoptera: Psocomorpha): Contrasting Rates in Mitochondrial Gene Rearrangement between Major Lineages of Psocodea
Source: PLoS One. 2013 Apr 22;8(4):e61685. doi: 10.1371/journal.pone.0061685 (PMC3632521; doi:10.1371/journal.pone.0061685)
Supplement: Table S1 — Primers used in the present study. (DOC) [file pone.0061685.s001.doc]

**Table S1: Primers used in the present** study

| **Fragment** | **Primer ID** | **Nucleotide sequence (5’-3’)** | **Reference** |
| --- | --- | --- | --- |
| 1 | TI-J34 | GCCTGATAAAAAGGRTTAYYTTGATA | [23] |
|  | C1-N1738 | TTTATTCGTGGRAATGCYATRTC | [23] |
| 2 | F1738 | ATGATTTCCTGCGGCTCTC | [23] |
|  | R2756 | GTTTAGGTTTCGGTCTGTGAG | [23] |
| 3 | C1-J2756 | ACATTTTTTCCTCAACATTT | [23] |
|  | C2-N3665 | CCACAAATTTCTGAACACTG | [23] |
| 4 | C2-J3399 | TCTATTGGTCATCAATGGTACTG | [23] |
|  | A8-N4061 | GAAAATAAATTTGTTATCATTTTCA | [23] |
| 5 | F4061 | AAGTAGACGCTAACCCTGGCCG | Present study |
|  | R8641 | GCACATGTAGAAGCTCCTGTAGC | Present study |
| 6 | N4-J8641 | CCAGAAGAACATAANCCRTG | [23] |
|  | N4L-N9629 | GTTTGTGAGGGWGYTTTRGG | [23] |
| 7 | F9629 | CTAACCCAAATAAAGGAGTC | Present study |
|  | R9648 | AGTAAAGAAACTGAGATAAGTAG | Present study |
| 8 | N4L-J9648 | TCCCAACACACCTTCACAAAC | [23] |
|  | CB-N11010 | TATCAACAGCAAATCCTCCTCA | [23] |
| 9 | F11010 | CCCGCAAATCCTTTAGTAAC | Present study |
|  | R12261 | AGCCAGGTCAGTTTCTATC | Present study |
| 10 | N1-J12261 | TACCTCATAAGAAATAGTTTGAGC | [23] |
|  | LR-N13000 | TTACCTTAGGGATAACAGCGTAA | [23] |
| 11 | LR-J12888 | CCGGTTTGAACTCARATCATGTAA | [23] |
|  | LR-N13889 | ATTTATTGTACCTTKTGTATCAG | [23] |
| 12 | F13889 | GCGGCTCTTTAAATCTTCAGTGA | Present study |
|  | R14197 | AGGAAACTTAAAGAATTTGGCGGTG | Present study |
| 13 | LR-J14197 | GTAAAYCTACTTTGTTACGACTT | [23] |
|  | SR-N14745 | GTGCCAGCAAYCGCGGTTATAC | [23] |
| 14 | F14745 | AGATAAGAGAATGACGGGCA | Present study |
|  | R34 | GCGATTGCTTGGATTAGAAAG | Present study |
