# Supplementary material for: Mitochondrial Genomes of Two Barklice, Psococerastis albimaculata and Longivalvus hyalospilus (Psocoptera: Psocomorpha): Contrasting Rates in Mitochondrial Gene Rearrangement between Major Lineages of Psocodea
Source: PLoS One. 2013 Apr 22;8(4):e61685. doi: 10.1371/journal.pone.0061685 (PMC3632521; doi:10.1371/journal.pone.0061685)
Supplement: Table S2 — Genes in the mitochondrial genome of the barklouse, Psococerastis albimaculata. (DOC) [file pone.0061685.s002.doc]

**Table S2. Genes in the mitochondrial genome of the barklouse, *Psococerastis albimaculata***

| **Gene** | **Direction** | **Location (bp)** | **Size (bp)** | **Anticodon** | **Start Codon** | **Stop Codon** | **Intergenic**  **Nucleotide^*^** |
| --- | --- | --- | --- | --- | --- | --- | --- |
| *trnM* | F | 1-65 | 65 | 33-35 CAT |  |  |  |
| *trnC* | R | 68-129 | 62 | 97-99 GCA |  |  | 2 |
| *trnI* | F | 126-190 | 65 | 155-157 GAT |  |  | -4 |
| *trnQ* | R | 188-252 | 65 | 221-223 TTG |  |  | -3 |
| *nad2* | F | 282-1274 | 993 |  | ATT | TAA | 29 |
| *trnW* | F | 1276-1340 | 65 | 1307-1309 TCA |  |  | 1 |
| *trnY* | R | 1343-1406 | 64 | 1374-1376 GTA |  |  | 2 |
| *cox1* | F | 1404-2939 | 1536 |  | ATT | TAA | -3 |
| *trnL2(UUR)* | F | 2935-2997 | 63 | 2964-2966 TAA |  |  | -5 |
| *cox2* | F | 2999-3683 | 685 |  | ATG | T- | 1 |
| *trnK* | F | 3684-3752 | 69 | 3715-3717 CTT |  |  | 0 |
| *trnD* | F | 3754-3819 | 66 | 3787-3789 GTC |  |  | 1 |
| *atp8* | F | 3820-3981 | 162 |  | ATA | TAA | 0 |
| *atp6* | F | 3975-4655 | 681 |  | ATG | TAA | -7 |
| *cox3* | F | 4655-5438 | 784 |  | ATG | T- | 3 |
| *trnG* | F | 5439-5500 | 62 | 5469-5471 TCC |  |  | 0 |
| *trnA* | F | 5501-5564 | 64 | 5530-5532 TGC |  |  | 0 |
| *trnR* | F | 5563-5628 | 66 | 5594-5596 TCG |  |  | -2 |
| *trnF* | R | 5631-5694 | 64 | 5660-5662 GAA |  |  | 2 |
| *nad5* | R | 5695-7414 | 1720 |  | ATA | T- | 0 |
| *nad3* | F | 7486-7839 | 354 |  | ATA | TAA | 71 |
| *trnN* | F | 7840-7904 | 65 | 7871-7873 GTT |  |  | 0 |
| *trnS1* | F | 7905-7971 | 67 | 7930-7932 GCT |  |  | 0 |
| *trnE* | F | 7971-8032 | 62 | 8002-8004 TTC |  |  | -1 |
| *trnH* | R | 8033-8095 | 63 | 8062-8064 GTG |  |  | 0 |
| *nad4* | R | 8099-9436 | 1338 |  | ATG | TAA | 3 |
| *nad4L* | R | 9430-9720 | 291 |  | ATT | TAA | -7 |
| *trnT* | F | 9722-9784 | 63 | 9753-9755 TGT |  |  | -16 |
| *trnP* | R | 9784-9849 | 66 | 9817-9819 TGG |  |  | -1 |
| *nad6* | F | 9851-10363 | 513 |  | ATT | TAA | 1 |
| *cytb* | F | 10363-11502 | 1138 |  | ATG | T- | -1 |
| *trnS2(UCN)* | F | 11501-11568 | 68 | 11531-11533 TGA |  |  | -2 |
| *nad1* | R | 11584-12516 | 933 |  | ATA | TAG | 15 |
| *trnL1(CUN)* | R | 12532-12596 | 65 | 12565-12567 TAG |  |  | 15 |
| *rrnL* | R | 12597-13836 | 1240 |  |  |  | 0 |
| *trnV* | R | 13837-13900 | 64 | 13867-13869 TAC |  |  | 0 |
| *rrnS* | R | 13901-14678 | 778 |  |  |  | 0 |
| *CR* |  | 14679-15589 | 911 |  |  |  | 0 |

*, negative numbers indicate that adjacent genes overlap.
