# Supplementary material for: Mitochondrial Genomes of Two Barklice, Psococerastis albimaculata and Longivalvus hyalospilus (Psocoptera: Psocomorpha): Contrasting Rates in Mitochondrial Gene Rearrangement between Major Lineages of Psocodea
Source: PLoS One. 2013 Apr 22;8(4):e61685. doi: 10.1371/journal.pone.0061685 (PMC3632521; doi:10.1371/journal.pone.0061685)
Supplement: Table S3 — Genes in the mitochondrial genome of the barklouse, Longivalvus hyalospilus. (DOC) [file pone.0061685.s003.doc]

**Table S3. Genes in the mitochondrial genome of the barklouse, *Longivalvus hyalospilus***

| **Gene** | **Direction** | **Location (bp)** | **Size (bp)** | **Anticodon** | **Start Codon** | **Stop Codon** | **Intergenic**  **Nucleotide^*^** |
| --- | --- | --- | --- | --- | --- | --- | --- |
| *trnC* | R | 1-60 | 60 | 28-30 GCA |  |  |  |
| *trnI* | F | 68-134 | 67 | 98-100 GAT |  |  | 7 |
| *trnQ* | R | 132-196 | 65 | 165-167 TTG |  |  | -3 |
| *nad2* | F | 235-1227 | 993 |  | ATT | TAA | 38 |
| *trnW* | F | 1235-1297 | 63 | 1265-1267 TCA |  |  | 7 |
| *trnY* | R | 1296-1357 | 62 | 1325-1327 GTA |  |  | -2 |
| *cox1* | F | 1355-2890 | 1536 |  | ATT | TAA | -3 |
| *trnL2(UUR)* | F | 2886-2949 | 64 | 2915-2917 TAA |  |  | -5 |
| *cox2* | F | 2951-3635 | 685 |  | ATG | T- | 1 |
| *trnK* | F | 3636-3702 | 67 | 3668-3670 CTT |  |  | 0 |
| *trnD* | F | 3705-3769 | 65 | 3737-3739 GTC |  |  | 2 |
| *atp8* | F | 3770-3928 | 159 |  | ATT | TAA | 0 |
| *atp6* | F | 3922-4602 | 681 |  | ATG | TAA | -7 |
| *cox3* | F | 4602-5385 | 784 |  | ATG | T- | -1 |
| *trnG* | F | 5386-5448 | 63 | 5416-5418 TCC |  |  | 0 |
| *trnA* | F | 5449-5512 | 64 | 5478-5480 TGC |  |  | 0 |
| *trnR* | F | 5511-5574 | 64 | 5542-5544 TCG |  |  | -2 |
| *trnF* | R | 5574-5639 | 66 | 5603-5605 GAA |  |  | -1 |
| *nad5* | R | 5640-7353 | 1714 |  | ATA | T- | 0 |
| *nad3* | F | 7427-7783 | 357 |  | ATT | TAA | 73 |
| *trnN* | F | 7784-7846 | 63 | 7814-7816 GTT |  |  | 0 |
| *trnS1(AGN)* | F | 7847-7912 | 66 | 7872-7874 GCT |  |  | 0 |
| *trnE* | F | 7912-7971 | 60 | 7942-7944 TTC |  |  | -1 |
| *trnH* | R | 7970-8031 | 62 | 7998-8000 GTG |  |  | -2 |
| *nad4* | R | 8038-9375 | 1338 |  | ATG | TAA | 6 |
| *nad4L* | R | 9369-9659 | 291 |  | ATT | TAA | -7 |
| *trnT* | F | 9661-9723 | 63 | 9692-9694 TGT |  |  | 1 |
| *trnP* | R | 9723-9786 | 64 | 9754-9756 TGG |  |  | -1 |
| *nad6* | F | 9788-10303 | 516 |  | ATT | TAA | 1 |
| *cytb* | F | 10296-11435 | 1140 |  | ATG | TAA | -8 |
| *trnS2(UCN)* | F | 11437-11504 | 68 | 11467-11469 TGA |  |  | 1 |
| *nad1* | R | 11531-12463 | 933 |  | ATA | TAG | 26 |
| *trnL1(CUN)* | R | 12479-12543 | 66 | 12512-12514 TAG |  |  | 15 |
| *rrnL* | R | 12544-13785 | 1241 |  |  |  | 0 |
| *trnV* | R | 13786-13850 | 65 | 13815-13817 TAC |  |  | 0 |
| *rrnS* (partial) | R | 13851-14442 | 592 |  |  |  | 0 |

*, negative numbers indicate that adjacent genes overlap.
