# Supplementary material for: Mitochondrial Genomes of Two Barklice, Psococerastis albimaculata and Longivalvus hyalospilus (Psocoptera: Psocomorpha): Contrasting Rates in Mitochondrial Gene Rearrangement between Major Lineages of Psocodea
Source: PLoS One. 2013 Apr 22;8(4):e61685. doi: 10.1371/journal.pone.0061685 (PMC3632521; doi:10.1371/journal.pone.0061685)
Supplement: Table S4 — Codon usage in the protein-coding genes of the mitochondrial genomes of the barklice, Psococerastis albimaculata and Longivalvus hyalospilus. (DOC) [file pone.0061685.s004.doc]

**Table S4. Codon usage in the protein-coding genes of the mitochondrial genomes of the barklice, *Psococerastis albimaculata* and *Longivalvus hyalospilus***

| **AA** | **Codon** | **N** |  | **%** |  | **AA** | **Codon** | **N** |  | **%** |  |
| --- | --- | --- | --- | --- | --- | --- | --- | --- | --- | --- | --- |
|  |  | **Pa** | **Lh** | **Pa** | **Lh** |  |  | **Pa** | **Lh** | **Pa** | **Lh** |
| F | **UUU** | **288** | **303** | **7.77** | **8.17** | Y | **UAU** | **127** | **150** | **3.43** | **4.05** |
| F | UUC | 70 | 64 | 1.89 | 1.73 | Y | UAC | 35 | 12 | 0.94 | 0.32 |
| L | **UUA** | **332** | **386** | **8.95** | **10.41** | ﹡ | **UAA** | **8** | **9** | **0.22** | **0.24** |
| L | UUG | 53 | 40 | 1.43 | 1.08 | ﹡ | UAG | 1 | 1 | 0.03 | 0.03 |
| L | CUU | 64 | 60 | 1.73 | 1.62 | H | **CAU** | **50** | **55** | **1.35** | **1.48** |
| L | CUC | 18 | 10 | 0.49 | 0.27 | H | CAC | 17 | 12 | 0.46 | 0.32 |
| L | **CUA** | **84** | **64** | **2.27** | **1.73** | Q | **CAA** | **58** | **62** | **1.56** | **1.67** |
| L | CUG | 16 | 3 | 0.43 | 0.08 | Q | CAG | 9 | 5 | 0.24 | 0.13 |
| I | **AUU** | **305** | **323** | **8.23** | **8.71** | N | **AAU** | **146** | **188** | **3.94** | **5.07** |
| I | AUC | 73 | 62 | 1.97 | 1.67 | N | AAC | 59 | 38 | 1.59 | 1.02 |
| M | **AUA** | **228** | **237** | **6.15** | **6.39** | K | **AAA** | **89** | **85** | **2.40** | **2.29** |
| M | AUG | 42 | 29 | 1.13 | 0.78 | K | AAG | 20 | 18 | 0.54 | 0.49 |
| V | **GUU** | **78** | **87** | **2.10** | **2.35** | D | **GAU** | **42** | **49** | **1.13** | **1.32** |
| V | GUC | 12 | 9 | 0.32 | 0.24 | D | GAC | 21 | 13 | 0.57 | 0.35 |
| V | GUA | 76 | 80 | 2.05 | 2.16 | E | **GAA** | **69** | **73** | **1.86** | **1.97** |
| V | GUG | 23 | 5 | 0.62 | 0.13 | E | GAG | 10 | 4 | 0.27 | 0.11 |
| S | **UCU** | **114** | **109** | **3.07** | **2.94** | C | **UGU** | **36** | **37** | **0.97** | **1.00** |
| S | UCC | 26 | 33 | 0.70 | 0.89 | C | UGC | 5 | 4 | 0.13 | 0.11 |
| S | UCA | 77 | 68 | 2.08 | 1.83 | W | **UGA** | **75** | **82** | **2.02** | **2.21** |
| S | UCG | 4 | 6 | 0.11 | 0.16 | W | UGG | 14 | 9 | 0.38 | 0.24 |
| P | **CCU** | **65** | **69** | **1.75** | **1.86** | R | CGU | 13 | 16 | 0.35 | 0.43 |
| P | CCC | 24 | 12 | 0.65 | 0.32 | R | CGC | 6 | 2 | 0.16 | 0.05 |
| P | CCA | 29 | 42 | 0.78 | 1.13 | R | **CGA** | **28** | **30** | **0.76** | **0.81** |
| P | CCG | 7 | 0 | 0.19 | 0 | R | CGG | 7 | 5 | 0.19 | 0.13 |
| T | ACU | 66 | 72 | 1.78 | 1.94 | S | AGU | 36 | 33 | 0.97 | 0.89 |
| T | ACC | 36 | 24 | 0.97 | 0.65 | S | AGC | 10 | 4 | 0.27 | 0.11 |
| T | **ACA** | **83** | **78** | **2.24** | **2.10** | S | **AGA** | **70** | **80** | **1.89** | **2.16** |
| T | ACG | 5 | 6 | 0.13 | 0.16 | S | AGG | 0 | 3 | 0 | 0.08 |
| A | **GCU** | **55** | **69** | **1.48** | **1.86** | G | GGU | 52 | 63 | 1.40 | 1.70 |
| A | GCC | 23 | 20 | 0.62 | 0.54 | G | GGC | 15 | 7 | 0.40 | 0.19 |
| A | GCA | 51 | 39 | 1.38 | 1.05 | G | **GGA** | **95** | **127** | **2.56** | **3.43** |
| A | GCG | 4 | 5 | 0.11 | 0.13 | G | GGG | 54 | 18 | 1.46 | 0.49 |

AA, amino acids; N, the number of times of a codon is used; %, the percentage of a codon in all used codons; ﹡, termination codon; Pa, *P. albimaculata*; Lh, *L. hyalospilus*.
